# A microfluidic platform for the characterisation of membrane active antimicrobials†
†Electronic supplementary information (ESI) available. See DOI: 10.1039/c8lc00932e


**DOI:** 10.1039/c8lc00932e

**Published:** 2019-01-30

**Authors:** K. Al Nahas, J. Cama, M. Schaich, K. Hammond, S. Deshpande, C. Dekker, M. G. Ryadnov, U. F. Keyser

**Affiliations:** a Cavendish Laboratory , Univ. of Cambridge , JJ Thomson Avenue , Cambridge CB3 0HE , UK . Email: ufk20@cam.ac.uk; b National Physical Laboratory , Hampton Road, Teddington , Middlesex TW11 0LW , UK; c Kavli Institute of Nanoscience , Delft Univ. of Technology , van der Maasweg 9 , Delft 2629 HZ , Netherlands

## Abstract

The spread of bacterial resistance against conventional antibiotics generates a great need for the discovery of novel antimicrobials. Polypeptide antibiotics constitute a promising class of antimicrobial agents that favour attack on bacterial membranes. However, efficient measurement platforms for evaluating their mechanisms of action in a systematic manner are lacking. Here we report an integrated lab-on-a-chip multilayer microfluidic platform to quantify the membranolytic efficacy of such antibiotics. The platform is a biomimetic vesicle-based screening assay, which generates giant unilamellar vesicles (GUVs) in physiologically relevant buffers on demand. Hundreds of these GUVs are individually immobilised downstream in physical traps connected to separate perfusion inlets that facilitate controlled antibiotic delivery. Antibiotic efficacy is expressed as a function of the time needed for an encapsulated dye to leak out of the GUVs as a result of antibiotic treatment. This proof-of-principle study probes the dose response of an archetypal polypeptide antibiotic cecropin B on GUVs mimicking bacterial membranes. The results of the study provide a foundation for engineering quantitative, high-throughput microfluidics devices for screening antibiotics.

## Abstract 2

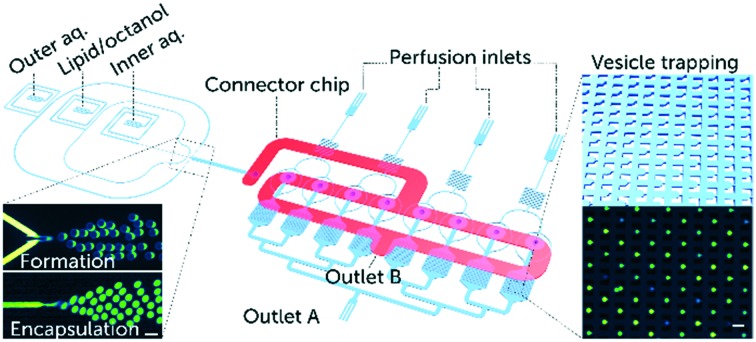
We quantify the membranolytic activity of antimicrobial peptides on biomimetic lipid vesicles in a multilayer microfluidic total analysis system.

## 1 Introduction

Phospholipid membranes provide bacterial cells with a permeability barrier that prevents conventional antibiotics from reaching their intracellular targets. This role of the membranes is particularly important for developing antibiotics targeting Gram-negative bacteria, whose two (outer and inner) membranes present a formidable challenge for any antibiotic to overcome.^1^,^2^ This is one major reason behind the failure of traditional target-oriented *in vitro* screening assays in the pharmaceutical industry, which repeatedly failed to discover novel agents active against Gram-negative bacteria simply because the candidate drugs failed to accumulate at sufficient, inhibitory concentrations in the vicinity of their targets. Therefore, there is a strong drive towards the discovery of antibiotics that target bacterial membranes.

To counteract microbial invasions, multicellular organisms rely on innate host defence mechanisms which make use of host defence peptides.^3^ These are effector molecules of innate immunity that have an intrinsic ability to recognise, bind to and disrupt microbial phospholipid membranes. This inspires continuous searches for novel peptides and attempts to design and redesign *de novo* engineered peptides into more effective antimicrobial agents. Naturally occurring, synthetic and designed antimicrobial peptides (AMPs) have evolved into one of the most promising classes of antimicrobial agents that can be converted into a sustainable pipeline of next generation antibiotics.^4^

However, an efficient pipeline requires effective tools to measure antimicrobial efficacy in a systematic manner ultimately opening up new possibilities for high-throughput screening platforms. Antimicrobial activity is typically measured by microdilution assays that provide the minimum inhibitory concentration (MIC) of a given antibiotic. The accuracy of such assays is subject to the inoculum effect,^5^ as the efficacy of AMPs may vary due to their stochastic binding to bacteria causing fluctuations in peptide concentration. Imaging techniques can reveal useful mechanistic information of the targeted activity at a single membrane level, which bulk culture assays, such as MIC assays, cannot capture. In this regard, highly controlled *in vitro* optical methods may provide drug developers with a highly efficient and reliable procedure for probing the antimicrobial activity of emerging antibiotics.

Most AMPs are cationic and favour interactions with anionic bacterial membranes. These interactions can be correlated with the phospholipid composition of the membranes allowing them to be reconstituted in empirically predictable model membranes.^6^ We leveraged this property of AMPs to develop a microfluidic assay, which quantifies the membranolytic activity of the peptides on thousands of individual, precisely defined model membranes. Membranes in the format of giant unilamellar vesicles (GUVs) are routinely used in research and drug screening including studies on drug permeation and transport through membrane pores,^7^–^10^ lipid scrambling^11^ and membrane fluctuation.^12^ The size of GUVs is compatible with the resolution of standard optical microscopy techniques which, in conjunction with microfluidic tools that can reliably form GUVs on chip,^13^–^16^ provide an attractive system for membranolytic screening. For our platform, we adapted the octanol-assisted liposome assembly (OLA) method as it has several advantages when compared to other on-chip possibilities. The technique yields homogeneous-size GUVs in a single step process coupled with a short vesicle maturation period (∼minutes), and enables the high-throughput production of vesicles.^15^

**Fig. 1:**
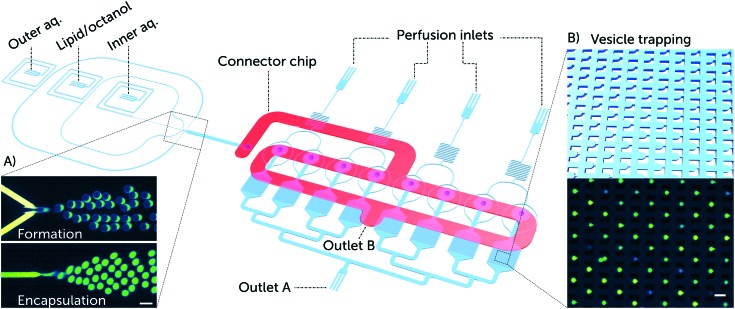
Overview of the microfluidic platform for testing the efficacy of membrane-active drugs on individual lipid vesicles. Schematic depiction of a complete lab-on-a-chip platform for vesicle formation, trapping, and drug testing. The platform consists of a vesicle formation component and 8 separate chambers each encompassing an array of 372 vesicle traps. Vesicle transport to the trap chambers is facilitated *via* a second layer of microfluidic channels, labelled as the connector chip (shown in red). The connector chip is a secondary PDMS layer that forms the only connection between the vesicle formation channels and the trap reservoirs. This is achieved by aligning the connector chip vertically above the punched fluidic outlet of the vesicle formation component and the 8 punched inlets of the trap reservoirs (ESI† Fig. S1). A) Microscopic fluorescence images of GUV formation using the octanol-assisted liposome assembly (OLA) technique. Vesicle formation is visualized with the fluorescent lipid Liss Rhod PE (560 nm/583 nm) labelling the lipid phase (top), and the encapsulation of the membrane impermeable HPTS dye (454 nm/520 nm) labelling the vesicle's interior (bottom). B) Schematic of vesicle trapping using an array of PDMS microposts as vesicle traps, and a corresponding microscopic fluorescence image of trapped GUVs encapsulating the HPTS dye. Scale bars represent 50 μm.

In order to optimise the output of a single experiment and minimise the consumption of valuable peptides required for characterisation, we developed a complete lab-on-a-chip platform that integrates the OLA vesicle formation component with a parallelised vesicle capture and drug perfusion system (Fig. 1). The device enables the preparation of GUVs with defined lipid compositions in abundant amounts with a rate of tens of Hz and at physiological salt concentrations.^15^ Thousands of GUVs can be immobilised downstream for long term studies. The trap chambers are connected to perfusion inlets, and the vesicles can be exposed to numerous different solutions in parallel (Fig. 1). The ability to investigate populations containing thousands of GUVs at the single-vesicle level is a considerable improvement on earlier techniques where studies were conducted on tens of GUVs per experiment in open chambers. Such throughput allows the acquisition of statistically significant results that portray the stochastic nature of lipid–peptide interactions.

We validate our platform for the characterisation of membrane active polypeptides by conducting a dose response study using cecropin B, a representative native AMP that is known to lyse Gram-negative bacterial cell membranes.^17^ Using our platform, we quantified the membranolytic activity of the peptide using a fluorescence-based readout that correlates peptide activity with the leakage of an encapsulated membrane-impermeable dye from the GUV. The validation is aimed at qualifying our approach as an AMP characterisation assay in the pre-clinical development of membrane active antibiotics.

## 2 Experimental

### 2.1 Microfluidic chip design

Our microfluidic platform incorporates an on-chip giant vesicle formation component.^19^ The octanol-assisted liposome assembly technique enables the production of double-emulsion droplets in a single step that result in giant unilamellar vesicles (Fig. 1A). The double emulsion droplets develop a pocket accumulating excess 1-octanol and lipids, which buds off on the timescale of minutes.^19^ We increased the size of the vesicles (typical size range 20–28 μm in diameter) and the volumetric flow rate by doubling the height and width of the six-way PDMS junction (design in the ESI† Fig. S1). The larger size of the GUVs aided their visualization and trapping, while the higher flow velocity optimised vesicle trapping, both in terms of speed and efficiency.

The vesicle immobilisation component of the platform contains 8 separate chambers of vesicle trap arrays inspired by a published trap design.^20^ The features of the trapping region were tuned to achieve efficient capture for vesicles with a diameter size of 20–25 μm. The gap size between the micro-posts of a single trap was designed to be 20–25% of the vesicles' diameter (design in the ESI† Fig. S2). Each trap array consists of 372 vesicle traps in a single chamber (Fig. 1B). All trap chambers are connected to perfusion inlets that are utilised for peptide delivery. The vesicle trap system enables us to conduct long duration studies of peptide–membrane interactions; our experiments typically lasted up to 10 hours post vesicle trapping.

The vesicle formation and capture components were integrated in a single device to perform the processes of vesicle formation and trapping sequentially and efficiently. To facilitate this, we designed a multi-layer device with a connector layer on top of the main layer, incorporating a 1 mm wide microfluidic channel. This optimises the transport and distribution of the vesicles into the trap arrays (Fig. 1). The connector chip offers four distinct advantages:

1. It provides a miniaturised logistical solution for the handling of giant vesicles formed in the device, as it circumvents the task of extracting the vesicles off-chip and re-introducing them into a second device for analysis.

2. It functions as a route for discharging vesicle production waste, and in the present design is used to remove excess 1-octanol from the solutions prior to vesicle immobilisation in the trap chambers.

3. It decouples the input pressures required for vesicle formation from the fluidics of vesicle trapping and drug perfusion. This is an important consideration, since vesicle formation is sensitive to small changes in input pressures.

4. It simplifies the surface treatment required for OLA vesicle formation.^15^ Before the assembly of the connector chip to the device, the vesicle formation and trapping components of the main bottom layer (Fig. 1) are separated. Thus only the vesicle formation part of the chip can be coated, with the trap chambers and perfusion system completely unaffected during the coating procedure. This precaution is important as the vesicle traps are rendered dysfunctional, if the material used for coating solidifies between the trapping micro-posts.^18^

### 2.2 Fabrication of the microfluidic chip

We fabricated a 3D microfluidic network in a multilayer polydimethylsiloxane (PDMS) device using standard photo- and soft-lithography techniques.^21^ Two master molds were fabricated for the bottom (main) and upper (connector) microfluidic layers (Fig. 1). The main channels were molded from a master with two distinct feature heights of ∼20 μm and ∼30 μm. The master mold of the connector channel had features of ∼40 μm in height. Silicon masters with feature heights around 20, 30 and 40 μm were generated by spin-coating (WS-650-23NPP, Laurell Technologies, USA) SU-8 2025 photoresist (Chestech, UK) at 3800, 2800, and 1800 rpm respectively for 60 s with a ramp of 100 rpm s^–1^ on 4′′ silicon wafers (University Wafer, USA). The spin coating was followed by a soft bake on a hot plate for 1 min at 65 °C followed by 5–6 min at 95 °C. A high-resolution, table-top laser direct imaging (LDI) system (LPKF ProtoLaser LDI, Germany) was used for prototyping on the resist-coated substrates directly using AutoCAD designs. In order to achieve different feature heights on the same silicon wafer, the photolithography process had to be performed twice on the same silicon wafer using an anchoring tool for aligning the features in the two designs. The printed master mold was then baked for 1 min at 65 °C followed by 5 min at 95 °C, developed in propylene glycol monomethyl ether acetate (PGMEA) for 2–3 min and hard-baked for 15 min at 125 °C.

A PDMS replica embedding the molded structures was obtained by mixing a siloxane elastomer with siloxane cross-linker known as the curing agent (Sylgard 184, Dow Corning) in a 9 : 1 ratio. The PDMS mixture was then degassed for 30 min in a desiccator, poured onto the SU-8 molds and baked in the oven for 90 min at 60 °C. After curing and peeling off the PDMS layers, inlets, outlets and connecting channels were punched with a 750 μm biopsy punch (WPI, UK) through the PDMS layers. The only exception was the outlet of the bottom main layer (outlet A), which was punched with a 1.5 mm biopsy punch. In order to accommodate the size of the microfluidic platform, glass slides with dimensions of 76 mm × 39 mm and 1 mm thickness were coated with PDMS and used as the base for the devices. The PDMS-coated glass slides were prepared by following a previous protocol.^15^ The main PDMS chips were bonded to the PDMS-coated glass slides by exposing the surfaces to an oxygen plasma (100 W plasma power, 10 s exposure, 25 sccm, plasma etcher from Diener Electric GmbH & Co. KG) and then annealing the two exposed surfaces together.

The surface of the microfluidic channels delivering the outer aqueous solution (OA) was treated for 15 min with polyvinyl alcohol (PVA) solution (50 mg mL^–1^, 87–90% hydrolysed, molecular weight 30 000–70 000 Da, Sigma-Aldrich).^19^ Post treatment, the PVA is removed *via* vacuum pump suction (Gardner Denver Thomas GmbH, Germany). A similar surface treatment was performed for the connector channel by manually painting the open channels with 10 μL of PVA (10 mg mL^–1^) using a pipette tip. The PVA-coated chips were then baked at 120 °C for 15 min. Finally, the connector PDMS layer was bonded on top of the main PDMS layer using a similar plasma treatment, while aligning the connector channel over the connecting holes (ESI† Fig. S3).

### 2.3 Vesicle formation

The OLA technique allows considerable variety in the content of the inner and outer aqueous phases (IA and OA). The base stock used for the IA and OA phases consisted of a 200 mM sucrose solution with 15% v/v glycerol in PBS for obtaining pH 7.4 and a physiological salt concentration. The OA additionally contained 50 mg mL^–1^ of Kolliphor P-188 (Sigma-Aldrich, UK), a poloxamer.^15^

Lipids were purchased from Sigma Aldrich in powder form and were dissolved in 100% ethanol to a final concentration of 100 mg mL^–1^. The lipid/octanol phase (LO) was prepared by diluting the lipid stock in 1-octanol to a 2–4 mg mL^–1^ concentration. A lipid mixture of 3 : 1 ratio 1,2-dioleoyl-*sn-glycero*-3-phosphocholine (DOPC) with 1,2-dioleoyl-*sn-glycero*-3-phospho-*rac*-(1-glycerol) sodium salt (DOPG) was used to mimic the bacterial membrane composition. In order to visualize the LO phase, a fluorescently labelled lipid (16 : 0 Liss Rhod PE, Avanti Lipids, 0.5 mg mL^–1^ in ethanol) was added to the LO phase. The pH sensitive dye HPTS, (8-hydroxypyrene-1,3,6-trisulfonic acid, Thermo Fisher), was used to stain the inner aqueous phase at a concentration of 50 μm. HPTS dye was chosen for this assay since it is a membrane impermeable molecule with high stability against photo-bleaching.^22^

The vesicle formation component was controlled with a pressure driven pump. We used a microfluidic flow control system MFCS-EZ (Fluigent GmbH, Germany) and its accompanying MAESFLOW software (version 3.2.1) to control the flow of the three different phases. The fluid reservoirs (Micrewtube 0.5 mL, Simport) were connected to the microfluidic chip *via* a polymer tubing (Tygon microbore tubing, 0.020′′ × 0.060′′ OD, Cole Parmer, UK) and a metal connector tip. Cut syringe needles (Gauge 23, BD Microbalance) or dispensing tips (Gauge 23 blunt end, Intertronics) served as connector tips between the tubing and the chip.

### 2.4 Cecropin B synthesis and dose preparation

Cecropin B (KWKVFKKIEKMGRNIRNGIVKAGPAIAVLGEAKAL-NH2) was assembled on a Liberty blue microwave peptide synthesiser (CEM) using standard Fmoc/*t*Bu solid-phase protocols with HBTU/DiPEA as coupling reagents on a Rink Amide MBHA resin (0.35 mmol substitution; 100 μmol scale). After post-synthesis cleavage and deprotection (95% TFA, 2.5% TIS, 2.5% water), the peptide was purified by semi-preparative reversed-phase high performance liquid chromatography (RP-HPLC). The identity of the peptide was confirmed by analytical RP-HPLC and MALDI-ToF. Analytical and semi-preparative RP-HPLC was performed on a Thermo Scientific™ Dionex™ HPLC system (Ultimate 3000), using Vydac C18 analytical and semi-preparative (both 5 μm) columns. Both analytical and semi-preparative runs used a 10–70% B gradient over 30 min at 1 mL min^–1^ and 4.5 mL min^–1^ respectively, with detection at 280 and 214 nm (buffer A, 5% and buffer B, 95% aqueous CH_3_CN, 0.1% TFA). Cecropin B was then lyophilised and stored in powder form at –80 °C. Small amounts of the lyophilised peptide to be tested were hydrated in Milli-Q water and used within two days of dissolution. The stock concentration was then measured using a NanoDrop™ system and diluted to 100 μM dilution in the IA buffer. The aliquot was then further diluted to the peptide concentrations required for the experiment. The perfusion of 2.5 or 5 μM of cecropin B solution into the microfluidic device required a minimal amount of 1–3 μg of the peptide in powder form. For visualization of drug arrival into the test chambers, we diluted the HPTS dye (5 μM) into the peptide solution. The peptide solutions were then stored at 4 °C while the stock was stored at –20 °C.

### 2.5 Device operation

After device fabrication and assembly, the chip was placed in a custom device holder (ESI† Fig. S3) that slots into a motor-driven automated Prior XYZ-stage attached to an Olympus IX73 inverted microscope. The fluids in the chip were controlled using 7 pressure ports and a single syringe pump module. Before connecting all the fluid reservoirs into the device, a critical step involves filling the trapping component with IA base stock to avoid air bubbles. This was done by connecting a neMESYS syringe pump module to outlet A (Fig. 1). The syringe was used to flow IA buffer at 50 μL h^–1^ through the trap chambers and push the air out of the connecting channels. Once this is complete, the flow rate was reduced to 3–5 μL h^–1^. The vesicle formation and perfusion inlets were then connected to the pressure pump reservoirs and positive fluid flows applied until an air-free device was obtained. At this stage, the reservoirs of the perfusion inlets were loaded with IA base stock. The outlet of the connector chip (outlet B) was then connected to a 2-SWITCH™ (Fluigent) that can switch between an open or closed configuration.

**Fig. 2:**
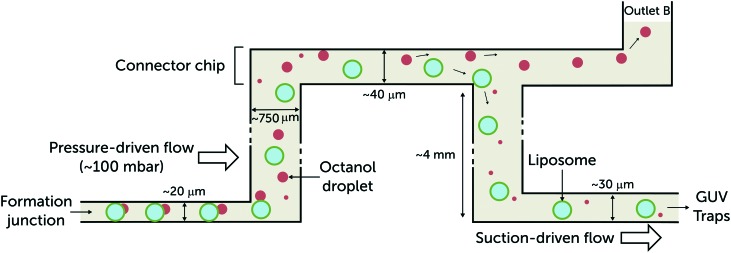
Side-view schematic illustrating vesicle transport through the multi-layer microfluidic device. The giant vesicles produced at the formation junction are accompanied by a 1-octanol pocket. The pocket buds off and forms a 1-octanol droplet that coexists with the vesicles. As they leave the formation channel, the 1-octanol droplets (*ρ* = 0.827 g mL^–1^) drift upwards and are separated.^18^ The vesicles are drawn into the vesicle trap arrays by applying suction at outlet A (Fig. 1). The height of the formation junction and post-formation channel is 20 μm. The vesicles are usually confined and in contact with the top and bottom walls of the channels until they arrive at the post-formation punched reservoir. In order to reintroduce the vesicles into microfluidic channels with minimum shearing, the heights of the channels after formation were designed to be larger than 20 μm : 40 μm for the connector chip and 30 μm for the trap region to avoid immobilising overlapping vesicles.

After formation, vesicles flow through a punched column to the connector chip (Fig. 2). During this stage, the 1-octanol pocket attached to a vesicle pinches off to form a 1-octanol droplet as a byproduct. The connector chip contains an outlet (outlet B in Fig. 1) that removes the vesicle production waste from the device based on density separation.^18^ The connector channel passes over the inlets of the connecting columns in order to distribute the vesicles into the trapping chambers. The trap chambers themselves are all connected to outlet A (Fig. 1). Trapping is achieved by applying suction at outlet A using the neMESYS syringe pump module. Fig. 2 schematically illustrates how the vesicles travel within the device. Once a majority of the vesicle traps have been filled, the suction tube is removed leaving outlet A open and the chambers are washed for an hour with IA buffer pumped from the perfusion inlets. The peptide dose was administered by filling the peptide-dye solutions into new reservoirs, replacing the initial reservoirs filled with IA buffer. The input pressure was maintained at ∼20 mbar throughout the measurements.

### 2.6 Data acquisition and image processing

The automated stage is synchronised with a Photometrics Evolve 512 camera *via* μManager 1.4 software with a wLS LED lamp from QImaging as the light source.^23^ A FITC filter cube set (Chroma) was used for tracking HPTS fluorescence. The automated stage enables the sequential acquisition of images from all the trapping chambers. The size of a single trapping chamber was designed to span 4 different fields of view when using a 10× air objective (Olympus UPLFLN). An acquisition cycle contains 32 selected positions that cover the 8 chambers, with a 30 second window between the first frame of every cycle. The acquisition was run over 2000 cycles, with the time-lapse images stored in TIFF format. During analysis, the trapped vesicles were manually selected using a custom plug-in of the software FIJI and the fluorescence intensities of the vesicles were measured.^24^ The acquired fluorescence signal trace for every vesicle was initially smoothed using a Savitzky–Golay filter in Origin. The signal was locally normalised to the highest initial intensity of the fluorescent vesicle coupled with global background subtraction using the signal after peptide-dye arrival. The single vesicle resolution data was sorted depending on the chamber that the vesicles were trapped in, which corresponded to the concentration of polypeptide tested. Since two trapping chambers were connected to a single perfusion inlet, four independent experiments could be run in parallel using one chip.

## 3 Results and discussion

In order to verify the assay, we monitored the vesicles' response towards different doses of the antimicrobial peptide cecropin B. At micromolar concentrations, the peptide has been observed to form pores in membranes leading to cell leakage.^25^,^26^ However, it has also been demonstrated that pores formed by AMPs are not limited to a particular diameter and can expand to the micron scale leading to the complete disintegration of the membrane.^27^

The effect of the AMP was observed by continuously exposing the trapped vesicles to 0, 2.5 and 5 μM of cecropin B in separate chambers. The concentrations were chosen similar to reported cecropin B MIC values against Gram-negative bacteria with MICs ranging between 0.15 and 4.2 μM.^28^,^29^ The viability of the trapped GUVs was monitored by recording the intensity of the HPTS dye, encapsulated in the vesicle interior, sequentially over 10 hours. The membrane disrupting activity of the polypeptides was quantified by correlating the fluorescent dye leakage to the integrity of the vesicle's lipid membrane. The time-point defining a compromised vesicle was set as the point when the fluorescence signal intensity decreased below 50% of its initial intensity.

**Fig. 3:**
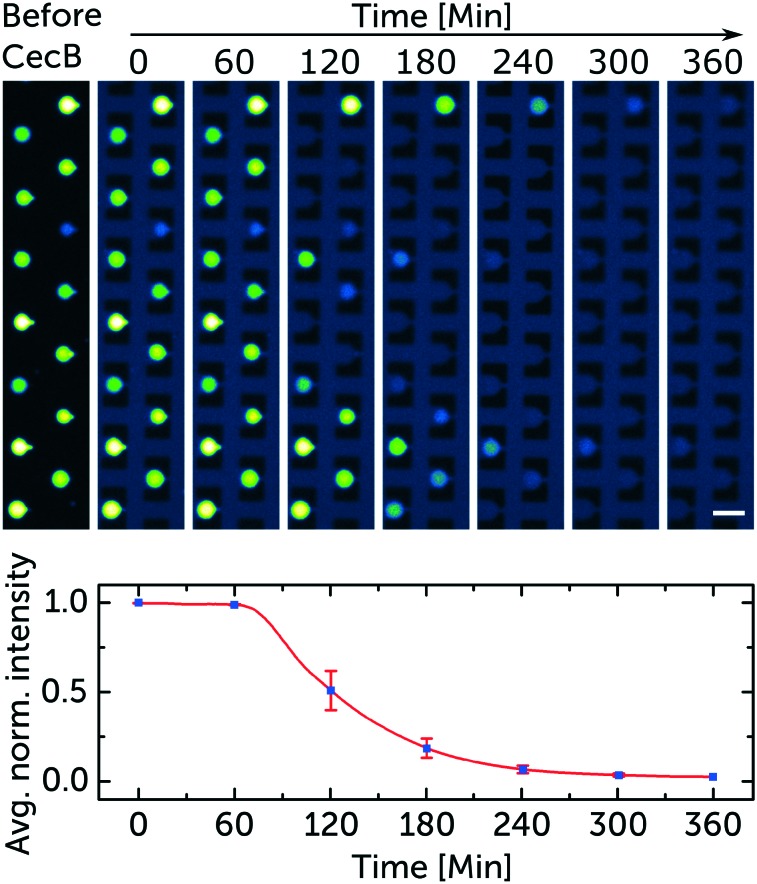
Representative sample of the data used to quantify the membranolytic activity of cecropin B. Fluorescence microscopy images of trapped GUVs (DOPC/DOPG lipids mixed at a 3 : 1 ratio). The trapped vesicles encapsulate the membrane impermeable dye HPTS (50 μM). The collage is a subset of images taken every hour displaying the time-dependent membranolytic effect of 5 μM cecropin B on the membrane integrity of the vesicles. The dark blue background marks the arrival of the peptide to the chamber. The averaged normalised fluorescence decrease in intensity from the 14 trapped vesicles is mapped. The means and standard error bars correspond to the fluorescence intensity of all the vesicles in the aligned frames in the collage. Scale bar represents 50 μm. The corresponding video is in the ESI† SV1.

Fig. 3 depicts how a representative group of trapped vesicles in one chamber behaves after exposure to 5 μM of cecropin B. Peptide arrival is displayed as a faint dark blue background colour, corresponding to the fluorescence of the diluted HPTS dye (5 μM) co-administered with the peptide solutions. The vesicle collage contains 8 subsets of vesicles at the same position with 1 hour in between each frame. The normalised intensity traces of the 14 trapped vesicles were averaged and plotted while displaying the standard error at the time points referencing the corresponding frames. Fig. 3 also shows some polydispersity in the trapped vesicles. This is typically caused either by small changes in the pressures applied to the lipid and aqueous phases during the vesicle formation process^19^ or by vesicle collisions with physical barriers.^30^ However, since our fluorescence measurement is relative to the initial intensity at the individual vesicle level, the polydispersity has no significant effect on the outcome of our assay.

**Fig. 4:**
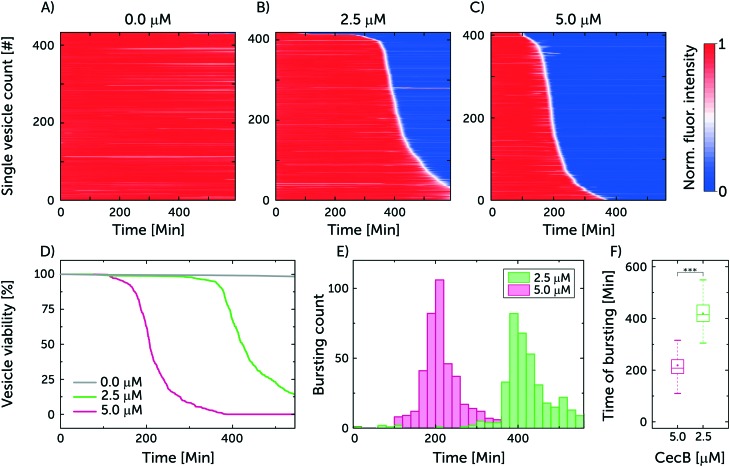
Cecropin B compromises model bacterial membranes in a dose-dependent manner. A–C) A summary of the membranolytic activity of 0 μM (control), 2.5 μM and 5 μM cecropin B on bacterial membrane-mimicking vesicles. Each horizontal line depicts the locally normalised intensity of encapsulated HPTS in a single trapped vesicle, with global background subtraction, over time. The vesicle's membrane is considered intact at high fluorescence intensity (red) and compromised at low fluorescent signal (blue). The intensity traces were ordered by the critical viability time point, which is defined as the point when the fluorescence signal intensity of a vesicle decreases below 50% of its initial intensity. Total number of analysed vesicles: *N*_A_ = 433, *N*_B_ = 419, *N*_C_ = 407. D) Depicts the vesicle viability over time based on the administered concentration of cecropin B. E) Event distribution of vesicle bursting and leakage as a function of drug concentration. F) Box plot depicting the time of bursting for the two cecropin B concentrations investigated. At 0.001 significance level, the Welch's *t*-test revealed statistically significant differences in the means of the two datasets.

Fig. 4 represents the results from a single experiment examining the population of vesicles at the single-vesicle level after treatment with cecropin B at 2.5 and 5 μM, in addition to a control sample without exposure to the polypeptide agent. In Fig. 4A–C every horizontal line depicts the normalised intensity of the encapsulated HPTS in a single vesicle. Red colour corresponds to the intensity of the encapsulated HPTS labelling intact vesicles, while the blue colour represents the reduced signal associated with the vesicle being compromised, due to dye leakage. The white colour represents the transition between the high and low intensities and describes the rate of the leakage process. Based on the rate of intensity decay, we can qualitatively infer whether the vesicles are porated (slow leakage) or burst (instant loss in intensity). However it is also possible that a certain number of intact but porated vesicles subsequently burst after dye leakage. Fig. 4D–F combines the results from the separate chambers to generate an overview describing the efficacy of cecropin B on the bacterial membrane mimics.

The viability of the vesicles untreated with peptide was observed to remain around 98% after 550 min. This confirms the integrity of untreated vesicles over the span of the experiment, and showcases the photostability of the encapsulated dye. On the other hand, the viability of vesicles treated with the higher 5 μM concentration of cecropin B deteriorated to 0% after 387.5 min of exposure while the lower 2.5 μM concentration led to a vesicle viability of 13% after 550 min. The bursting events correspond to damage in the vesicle's integrity either through leakage or complete vesicle lysis. In the chambers with 2.5 μM, the mean time point for vesicle bursting was 419.4 ± 3.4 min (*N* = 419, mean ± s.e.m.) while in the case of 5 μM it was 218.3 ± 2.7 min (*N* = 407, mean ± s.e.m.). Thus doubling the peptide concentration effectively halved the vesicle bursting time, as expected for antimicrobial agents that cooperatively assemble in bacterial membranes.^31^

Three repeats were conducted using the previous conditions. The cumulative sum of the vesicles in the three repeats for 0, 2.5 and 5 μM of cecropin B was 1042, 1103 and 1350 respectively. The viability of the vesicles for the control after 550 min was 98 ± 0.3% while the mean time point (mean ± s.e.m.) for vesicles bursting at 2.5 and 5 μM was 392.5 ± 2.8 min and 211.2 ± 2.2 min, respectively (independent repeats can be found in ESI† Fig. S5 and S6).

## 4 Conclusions

We have designed a fully integrated microfluidic platform to test the efficacy of AMPs on biomimetic vesicle membranes. GUVs can be produced on demand, encapsulating a membrane impermeable dye as an integrity marker. AMP activity is quantified by studying the time taken for the vesicles to lyse or porate, which is accompanied by dye leakage and a corresponding decrease in vesicle fluorescence. The vesicles are immobilised for long duration studies and can be controllably washed and exposed to membrane active molecules. We validated this assay by studying the time-dependent activity of the native antimicrobial peptide cecropin B on bacterial mimicking model membranes. This standardised platform can now be used to quantify the activity of any AMPs, either designed or native, that lyse or porate lipid membranes. Our platform allows us to study over 1000 vesicles simultaneously per experiment. The vesicles were continuously perfused with a defined peptide concentration for the duration of the assay. Cecropin B was found to disrupt negatively charged vesicle membranes in a dose-dependent manner, consistent with the cooperative assembly of AMPs in membranes typically accompanied with pore formation. The cecropin B concentrations we tested are in the range of reported MIC values, thus proving that our assay yields data comparable to results from traditional biological assays.^28^,^29^ On the other hand, the untreated vesicle population showed only a 2% loss in viability throughout the experiment, ensuring the stability of the vesicles in the trap chambers.

Our lab-on-a-chip platform can be utilised in a wide range of biophysical applications and systematic studies that target lipid membranes. The ability to controllably change the lipid composition^19^ allows us to mimic bacterial or mammalian cell membranes for studying the membranolytic and hemolytic activities of novel peptides. We can thus use this device to study both the efficacy and selectivity of antimicrobial peptides in a quantitative, controlled manner as well as provide mechanistic insights into their mode of action. The microfluidic platform is tailored for drug development since it uses minimal quantities of potentially expensive drug candidates in comparison to bulk assays. Additionally, as demonstrated with the parallel trap chambers, the assay can be further parallelised to test multiple different drugs or drug concentrations in the same experiment. We envisage this assay as a new biometrological standard for the pre-clinical development of membrane-active polypeptide antimicrobials.

## Conflicts of interest

There are no conflicts to declare.

## Supplementary Material

Supplementary information

Supplementary movie
