# Supplementary material for: A microfluidic platform for the characterisation of membrane active antimicrobials
Source: Lab Chip. 2019 Jan 30;19(5):837–44. doi: 10.1039/c8lc00932e (PMC6404476; doi:10.1039/c8lc00932e)
Supplement: Supplementary file 1 [file LC-019-C8LC00932E-s001.pdf]

This journal is © The Royal Society of Chemistry 2018

Supplementary information for:

A Microfluidic Platform for the Characterisation of Membrane Active Antimicrobials

K. Al Nahas, J. Cama, M. Schaich, K. Hammond, S. Deshpande, C. Dekker, M. G. Ryadnov and U. F. Keyser

Supplementary Figure S1.

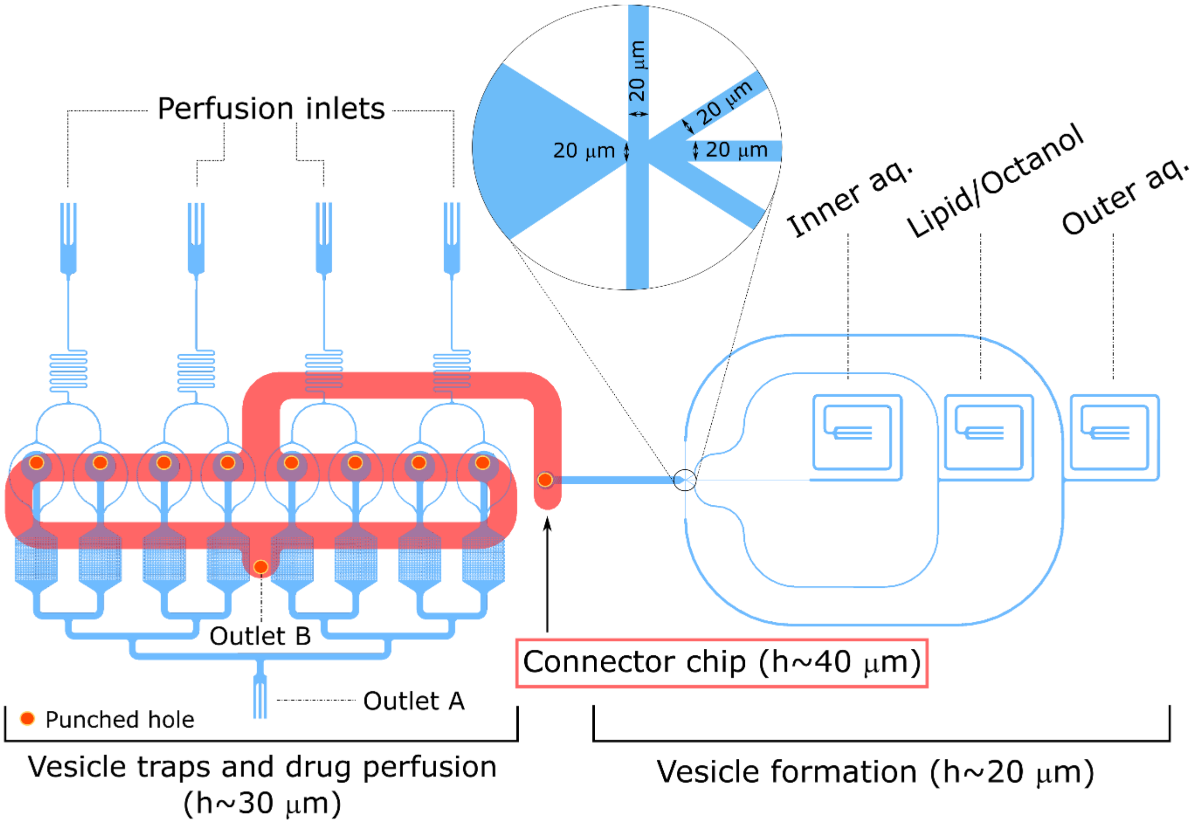

Schematic depiction of the microfluidic chip design. The design has a fingerprint spanning 19.1 x 42.5 mm. The IA, LO and OA channels of the vesicle formation component all have widths of 20 µm at the vesicle formation junction. The post-vesicle formation channel has dimensions of 0.3 x 4.6 mm until it reaches the connecting punched hole. The connector chip is 1 mm wide in order to easily align it with the ⌀ 0.75 mm connecting punched holes. The perfusion channels are 60 µm wide, and each inlet can feed 2 trapping chambers with the same solution. The vesicle seeding channels, 250 µm in width, are flanked by 2 perfusion channels, one on each side. The trap chambers are all connected to a single outlet. The approximate heights of the different sections of the device are indicated within the figure.

Supplementary Figure S2.

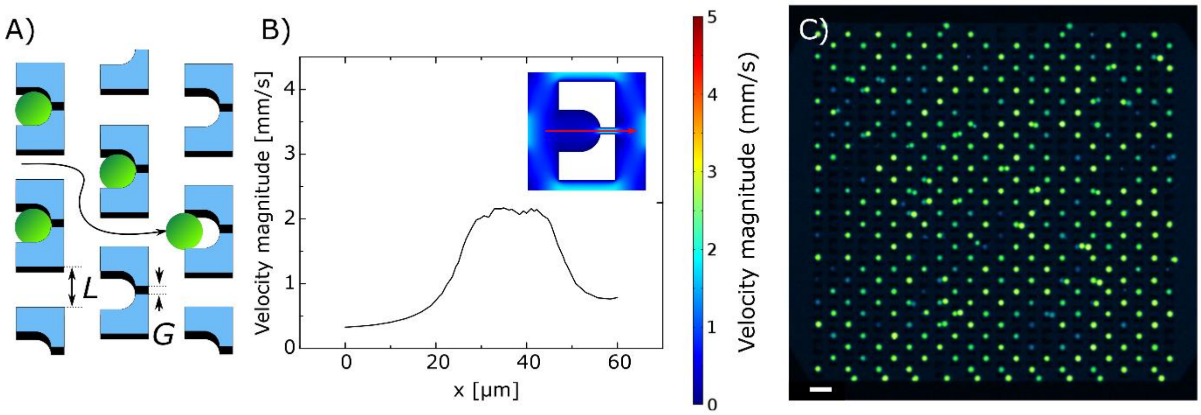

(A) Schematic design of the vesicle traps adapted from Kazayama *et al*. (2016), optimised to trap vesicles with sizes between ⌀ 20-25 μm. The passages between traps [L] are 26 μm in width while the gap within the vesicle traps [G] is 5 μm. A trap array consists of 24 rows of traps; the rows alternate between 15 and 16 traps each and consecutive rows are offset to enhance trapping efficiency. (B) The fluid flow profiles in and around the traps were simulated using finite element methods (COMSOL Multiphysics 4.4), the x-axis corresponds to the flow profile along the displayed red arrow. The flow profile indicates that the design is able to attract flowing vesicles but is not high enough to cause shearing stress. (C) Fluorescence microscopy image (4x air obj. Olympus UPLFLN) of a trap chamber after capturing GUVs encapsulating the membrane impermeable HPTS dye (454 nm/520 nm). The trapped vesicles show some polydispersity in intensity as a result of: (i) changes in the applied pressures on the lipid and aqueous phases during the vesicle formation process and (ii) vesicle splitting post-formation after collision with a physical barrier or due to any shear stress. The scale bar represents 100 μm.

Supplementary Figure S3.

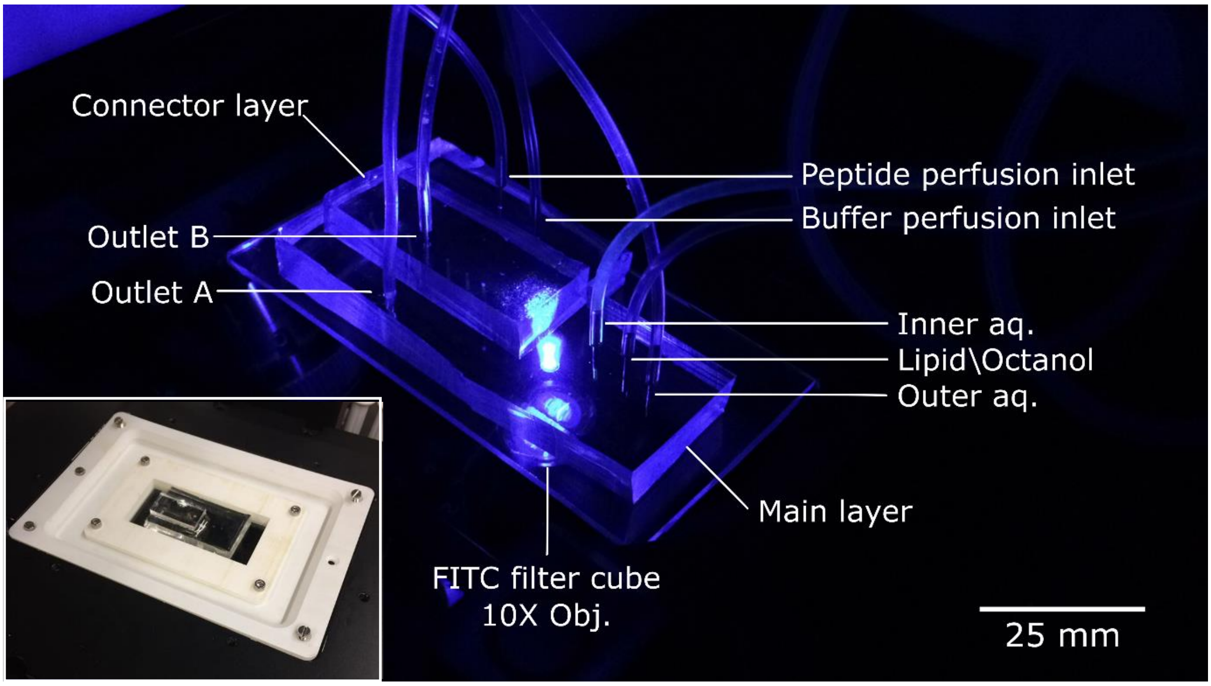

Image of the microfluidic device mounted on an Olympus IX73 epifluorescence microscope, with fluid tubes connected and labelled in correspondence to their contents. Inset image displays a custom 3D printed device holder.

Supplementary Figure S4.

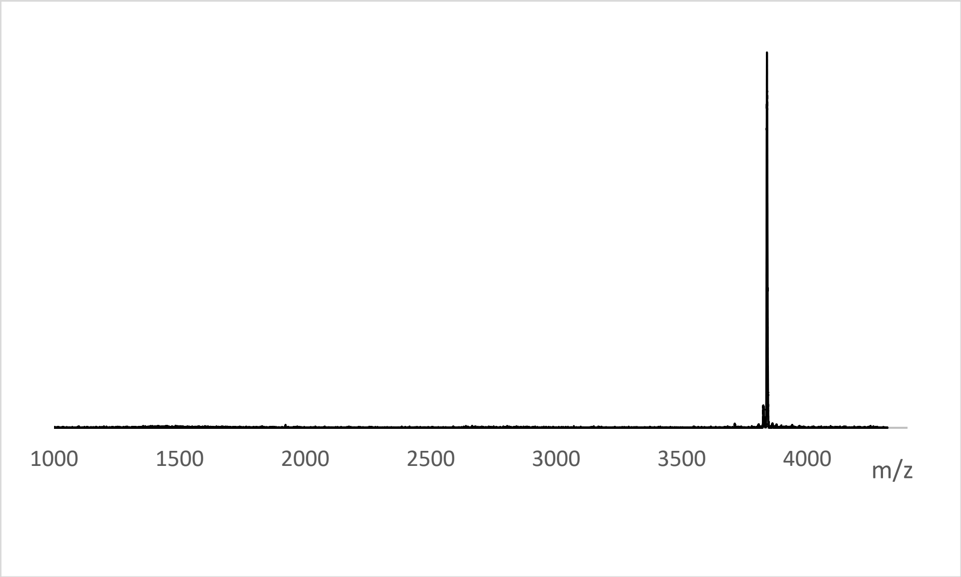

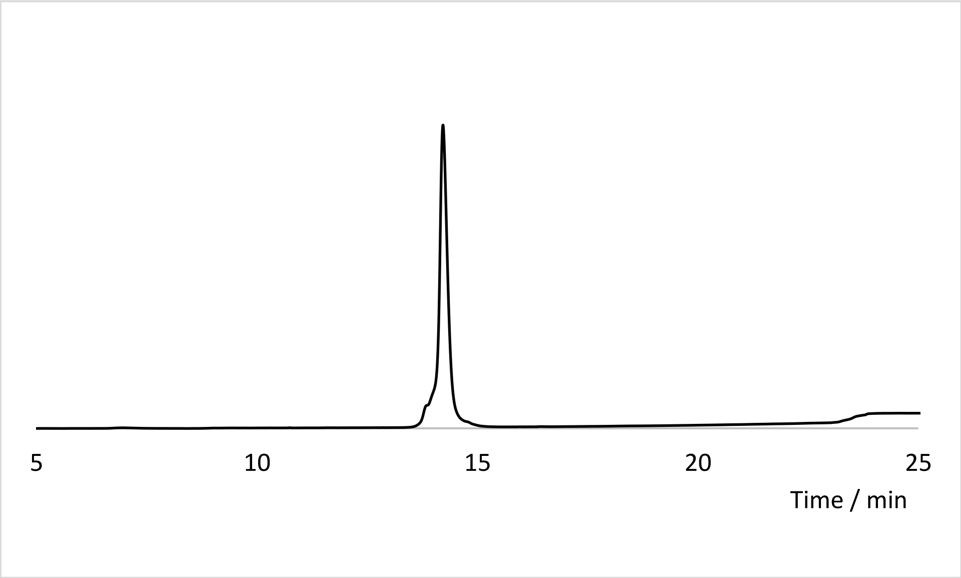

For cecropin B, the peptide was purified by semi-preparative reversed-phase high performance liquid chromatography (RP-HPLC). The identity of the peptide was confirmed by analytical RP-HPLC and MALDI-ToF. MS [M+H]+: m/z 3834.37 (calc) 3838.1 (found). Analytical and semi-preparative RP-HPLC was performed on a Thermo Scientific™ Dionex™ HPLC system (Ultimate 3000), using a Vydac C18 analytical and semi-preparative (both 5 µm) column. Both analytical and semi-preparative runs used a 10-70% B gradient over 30mins at 1 mL/min and 4.5 mL/min respectively, with detection at 280 and 214 nm. (Buffer A, 5% and buffer B, 95% aqueous CH3CN, 0.1% TFA).

Supplementary Figure S5.

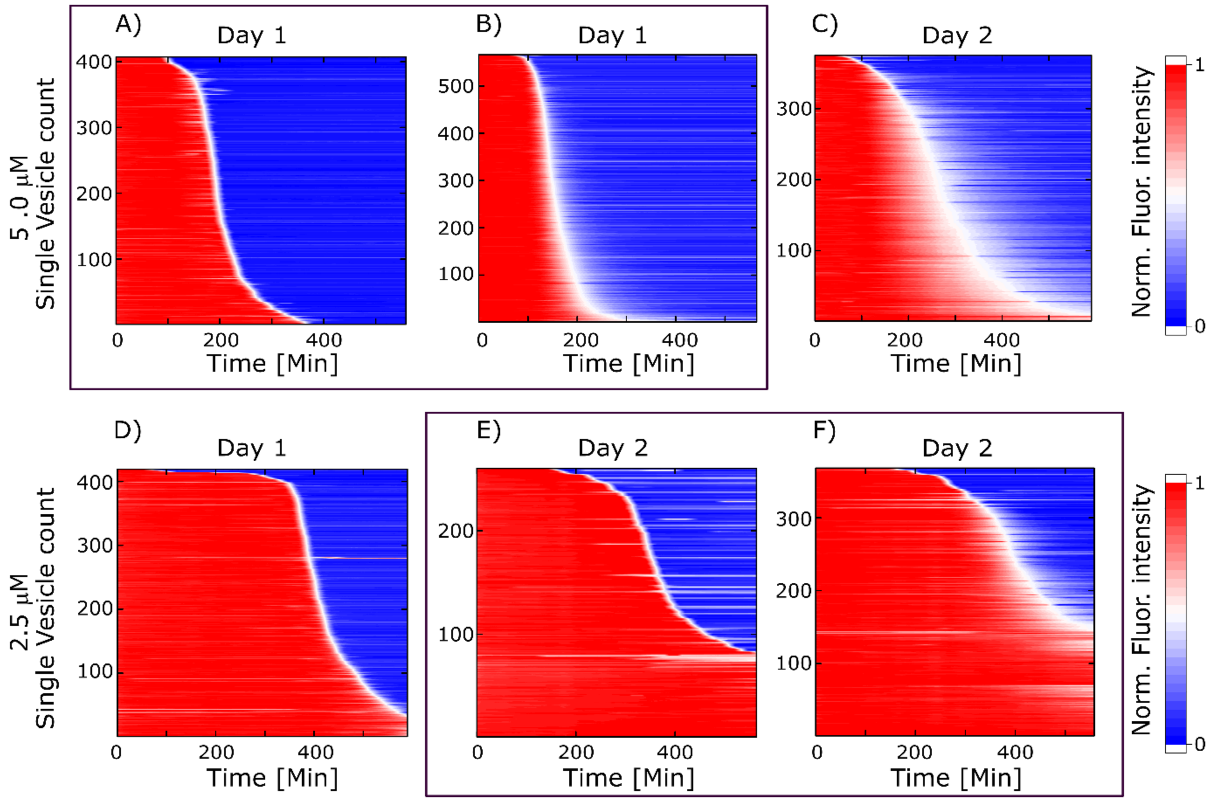

Three independent dose response analysis experiments with cecropin B at 5 µM (A-C) and 2.5 µM (D-E) acting on trapped GUVs (3:1 ratio of DOPC:DOPG lipids). Each horizontal line depicts the locally normalized intensity of encapsulated HPTS in a single trapped vesicle, with global background subtraction, over time. The vesicle's membrane is considered intact at high fluorescence intensity (Red) and compromised at low fluorescent signal (Blue). The intensity traces were ordered by the critical viability time point, which is defined as the point when the fluorescence signal intensity of a vesicle decreases below 50% of its initial intensity. These experiments were conducted with the same preparatory conditions in three different devices; the only distinction is in the time frame of using the cecropin B after dissolution from its lyophilized state. The Day 1 studies show the experiment when the peptide was dissolved and used on the same day (A, B and D), while the Day 2 studies show the experiments when the dissolved stock (100 µM) of cecropin B was used after storage for 1 day in the fridge at 4 ^{o}C (C, E and F). We observed a clear decline in the peptide's membranolytic activity after overnight storage at 4 ^{o}C in dissolved form. This shows that peptide efficacy is critically dependent on its handling and storage. This also shows that our experiments are sensitive to such effects. The assay can therefore also be used to study peptide aging and its correlation with efficacy. However, for future standardised testing on novel AMPs, our results suggest that one should always test the peptide on the day that the dissolution was performed.

Supplementary Figure S6.

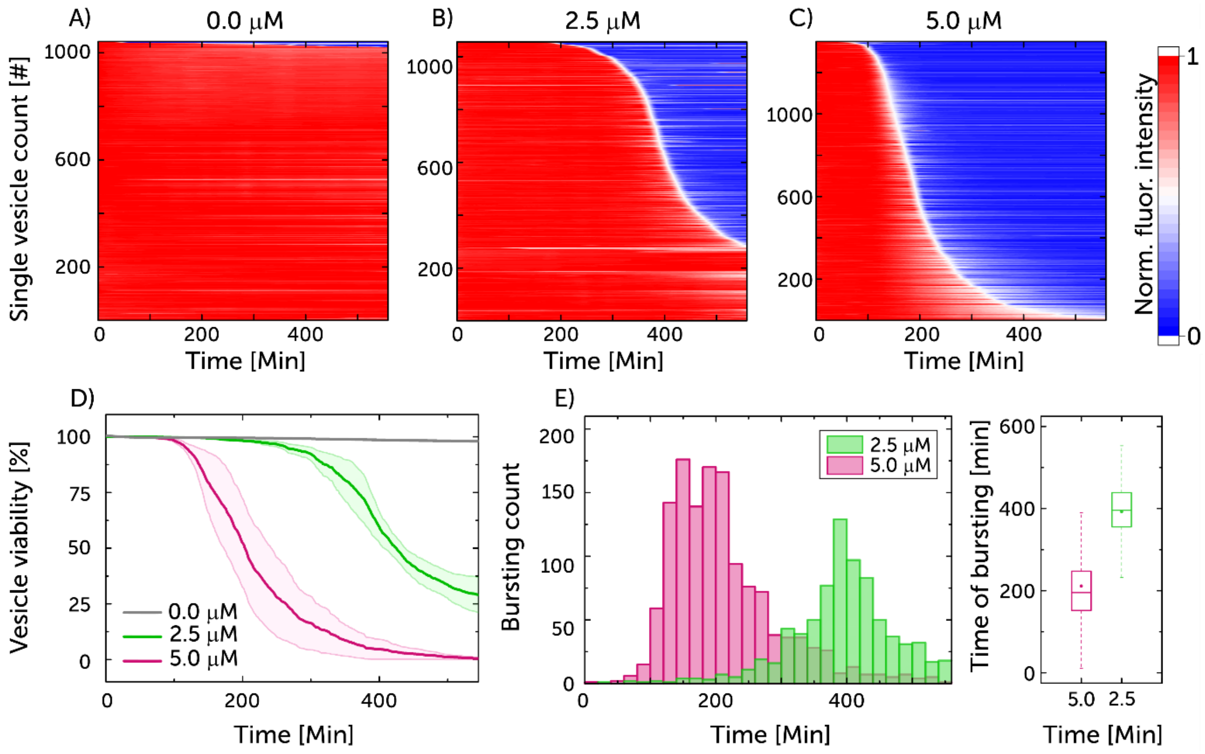

Combined summary of cecropin B's dosage analysis from the three independent repeats (mentioned in Fig. S5). A-C) Combined summary of the membranolytic activity of 0 μM (Control), 2.5 μM and 5 μM cecropin B on bacterial mimicking vesicles. Total number of analysed vesicles: N_A = 1042, N_B = 1103, N_C = 1350. D) Depicts the vesicle viability over time based on the administered concentration of cecropin B with shaded standard error. E) Event distribution of vesicle bursting and leakage as a function of drug concentration. F) Box plot depicting the time of bursting for the two cecropin B concentrations investigated. At 0.001 significance level, the Welch's t-test revealed statistically significant differences in the means of the two datasets. The viability of vesicles treated with 5 μM concentration of cecropin B deteriorated to 0.4 ± 0.4 % by the end of the 550 min of exposure while the lower 2.5 μM concentration resulted in a vesicle viability of 28.4 ± 8 %.

Supplementary Video SV1.

The supplementary video in AVI format displays the membranolytic activity of cecropin B over a span of 450 minutes, with frames 30 seconds apart. The video displays 14 trapped vesicles encapsulating the HPTS dye and exposed to 5 μM of cecropin B, as shown in Fig. 3 in the main text. The membranolytic activity of the peptide is inferred via the leakage of the dye from the GUV via poration of the membrane, which is observed in the video as a drop in the internal fluorescence of the GUVs.
